# A phase I study of the safety and tolerability of olaparib (AZD2281, KU0059436) and dacarbazine in patients with advanced solid tumours

**DOI:** 10.1038/bjc.2011.8

**Published:** 2011-02-15

**Authors:** O A Khan, M Gore, P Lorigan, J Stone, A Greystoke, W Burke, J Carmichael, A J Watson, G McGown, M Thorncroft, G P Margison, R Califano, J Larkin, S Wellman, M R Middleton

**Affiliations:** 1 University of Oxford Department of Oncology, Churchill Hospital, Old Road, Oxford OX3 7LJ, UK; 2 Royal Marsden Hospital, London SW3 6JJ, UK; 3 Department of Medical Oncology, Christie Hospital, Manchester M20 4BX, UK; 4 KuDOS Pharmaceuticals, 410 Cambridge Science Park, Cambridge CB4 0PE, UK; 5 Cancer Research UK Carcinogenesis Group, Paterson Institute for Cancer Research, Manchester M20 9BX, UK; 6 AstraZeneca, Alderley Park, Macclesfield SK10 4TG, UK

**Keywords:** chemotherapy, dacarbazine, melanoma, PARP, resistance

## Abstract

**Background:**

Poly adenosine diphosphate (ADP)-ribose polymerase (PARP) is essential in cellular processing of DNA damage via the base excision repair pathway (BER). The PARP inhibition can be directly cytotoxic to tumour cells and augments the anti-tumour effects of DNA-damaging agents. This study evaluated the optimally tolerated dose of olaparib (4-(3--4-fluorophenyl) methyl-1(2H)-one; AZD2281, KU0059436), a potent PARP inhibitor, with dacarbazine and assessed safety, toxicity, clinical pharmacokinetics and efficacy of combination treatment.

**Patients and methods:**

Patients with advanced cancer received olaparib (20–200 mg PO) on days 1–7 with dacarbazine (600–800 mg m^−2^ IV) on day 1 (cycle 2, day 2) of a 21-day cycle. An expansion cohort of chemonaive melanoma patients was treated at an optimally tolerated dose. The BER enzyme, methylpurine-DNA glycosylase and its substrate 7-methylguanine were quantified in peripheral blood mononuclear cells.

**Results:**

The optimal combination to proceed to phase II was defined as 100 mg bd olaparib with 600 mg m^−2^ dacarbazine. Dose-limiting toxicities were neutropaenia and thrombocytopaenia. There were two partial responses, both in patients with melanoma.

**Conclusion:**

This study defined a tolerable dose of olaparib in combination with dacarbazine, but there were no responses in chemonaive melanoma patients, demonstrating no clinical advantage over single-agent dacarbazine at these doses.

One of the primary roles of poly adenosine diphosphate (ADP)-ribose polymerase-1 (PARP-1) is to detect DNA strand breaks that occur following DNA damage. Activation of PARP-1 in this setting results in DNA repair via the base excision repair pathway (BER) (Virag and Szabo, 2002). The PARP-1 activity is frequently increased in tumour cells, with evidence that inhibition of PARP can be cytotoxic (Chalmers, 2009). The PARP inhibition leads to accumulation of DNA single-strand breaks resulting in DNA double-strand breaks at replication forks. The PARP-1 inhibition augments the anti-tumour effects of many DNA-damaging cytotoxic agents or radiation (Chalmers, 2009).

Olaparib (4-(3--4-fluorophenyl) methyl-1(2H)-one; AZD2281) is a potent, orally active, PARP inhibitor (Menear *et al*, 2008). Olaparib combined with the methylating agent temozolomide, a pro-drug of 3-methyl-(triazen-1-yl-4-carboxamide (MTIC), enhances cytotoxicity in tumour cell lines and xenografts (Menear *et al*, 2008). Dacarbazine, dimethyltriazenoimidazole carboxamide (DTIC) which is also activated to form MTIC, was selected for clinical investigation in combination with olaparib to explore the combination's activity in melanoma.

DNA adducts, such as 3-methyladenine (3meA) and *N*7-methylguanine (7-meG), that are produced in DNA following exposure to dacarbazine are processed by the BER pathway (Fromme *et al*, 2004). A damage-specific DNA glycosylase removes the alkylated DNA base, resulting in the generation of apurinic sites that are cleaved by the abundant endonuclease, APE, and the resulting single-strand breaks are repaired by the coordinated intervention of PARP, DNA polymerase *β*, X-ray repair cross-complementing-1 and ligases I and III (Dantzer *et al*, 1999). The PARP senses and is activated by the DNA single strand breaks and catalyses poly ADP ribosylation and consequent activation of the various proteins involved in the BER pathway.

The aim of combining dacarbazine with olaparib was to disrupt BER function, and thus elicit an accumulation of strand breaks and ultimately increased cytotoxicity. Given the requirement of single-strand break formation in this process, PARP inhibition may have a greater impact in cells with a greater ability to remove the damaged bases, that is, with higher *N-*methylpurine-DNA glycosylase (MPG) activity. This activity may itself be upregulated by combination treatment with a PARP inhibitor and an alkylating agent, and this may have consequences for cell death, as 3meA is a toxic DNA lesion (Tentori *et al*, 1999, 2001).

The rationale for this study (KU36-73, NCT00516802) was that intermittent dosing of olaparib, given in combination with dacarbazine, would sufficiently inhibit the PARP-1 repair of methylated DNA to produce an enhanced clinical effect. Primary objectives were to determine the safety, tolerability and dose-limiting toxicity (DLT) of a combination of oral olaparib and intravenous dacarbazine in patients with advanced solid tumours. Secondary objectives were to determine the pharmacokinetic profile of olaparib in combination with dacarbazine, to investigate the pharmacokinetic–pharmacodynamic profile of the combination in surrogate tissues and to enable a preliminary assessment of their anti-tumour activity. Thus both inter- and intraindividual variations in MPG activity during the course of the KU-DTIC treatment cycle were measured. Levels of 7-meG in DNA were quantified before treatment and following DTIC as both an indicator of the level of alkylation damage generated and an indicator of the amounts lost through repair or cell turnover, respectively.

## Patients and methods

The study was conducted in accordance with the Principles of the International Conference on Harmonization of Good Clinical Practice guidelines and the Declaration of Helsinki. The protocol was approved by an independent ethics committee, according to UK and local requirements. All patients enroled in the study gave informed written consent (KU36-73, NCT00516802).

Individuals aged ⩾18 years with a life expectancy of at least 3 months were eligible for the study. The ECOG performance status of 2 or better, adequate hepatic, renal and bone marrow function, and platelet count were required. For the initial dose-escalation cohorts, patients had to have a histologically or cytologically confirmed malignant solid tumour refractory to standard therapy. At a dacarbazine dose of 800 mg m^−2^ in the escalation phase and in the dose-expansion phase, only patients with unresectable stage III/IV cutaneous or unknown primary melanoma and no previous systemic cytotoxic chemotherapy were allowed to participate.

### Treatment

Olaparib (AstraZeneca, Macclesfield, UK) was administered orally as capsules twice daily on days 1–7 of each 21-day treatment cycle. In cycle 2, olaparib was administered on days 2–8 to permit the pharmacokinetics of dacarbazine given alone to be determined. Dacarbazine (Bayer, Bedford, UK) was administered by intravenous infusion over 60 min on day 1 of each cycle, 3 h after olaparib administration (except in cycle 2).

### Study design

**Figure 1:**
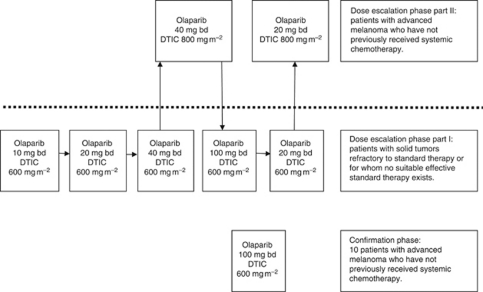
Chronological flow chart of implemented study design cohorts.

The starting dose was olaparib 10 mg bd with 600 mg m^−2^ dacarbazine (10: 600: combination doses will be abbreviated in this way for the remainder of the manuscript). The dose for successive cohorts was modified according to the scheme presented in Figure 1 taking into account the toxicities experienced by preceding patient cohorts.

The DLT was defined as any of the following: grade 4 haematological toxicity lasting ⩾5 days; grade 3 or 4 febrile neutropaenia; and grade 3 or 4 non-haematological toxicity. However, grade 3 or greater non-haematological toxicities were not classified as DLTs, if the nature and severity of the toxicity was attributable to DTIC alone. If one out of three patients at a dose level developed DLT, up to three additional patients were treated at that dose level. If one out of the additional patients developed DLT, dose escalation ceased and a preceding dose level or an intermediate dose level was tested. This lower dose was defined as the maximum tolerated dose for that dose of dacarbazine, unless ⩾2 out of 6 patients developed DLT.

The dose-expansion phase involved 10 patients treated at the selected olaparib and dacarbazine doses, with the option to move to a randomised expansion phase, if a 20% overall response rate was observed.

### Toxicity and response evaluation

Safety assessments included physical examination, chemistry, haematology and urinalysis. Toxicities were evaluated at least weekly during the study period and were graded according to the National Cancer Institute Common Toxicity Criteria version 3 (Tsuchida and Therasse, 2001). Efficacy was assessed every other cycle.

### Pharmacodynamics

The MPG activity and levels of 7-meG in DNA were determined in peripheral blood mononuclear cells before and following treatment with dacarbazine. Samples were obtained on days 1 and 8 of the treatment cycle, and analysed according to previously published methods. The MPG activity in the cell extract was quantified by an oligonucleotide cleavage assay. The amounts of 7-meG in peripheral blood DNA were quantified according to an immunoblot method (Harrison *et al*, 2001).

### *N-*methylpurine-DNA glycosylase activity

Whole blood (1 ml) was thawed, centrifuged at 3700 **g** for 10 min and the supernatant discarded. The cell pellet was resuspended in buffer, and extracts prepared by sonication as described by Harrison *et al* (2001). The MPG activity in the cell extract was quantified by an oligonucleotide cleavage assay. Briefly, an oligonucleotide containing ethenoadenine (a substrate for MPG) close to the 5′-end was labelled with ^32^P *γ*ATP, annealed to its 5′-biotinylated complement and immobilised on a streptavidin-coated 96-well plate. Incrementally increasing amounts of cell extract based on DNA content as quantified by picogreen-based assay (Invitrogen Ltd., Paisley, UK) were incubated with the substrate oligonucleotide for 3 h at 37°C. The ^32^P-labelled 5-mer released into the supernatant as a result of removal of ethenoadenine by MPG, and the subsequent action of APE were quantified on a TOP COUNT machine (Perkin Elmer LAS, Beaconsfield, UK). Specific activity was calculated as femtomoles (Fmoles) ethenoadenine removed per *μ*g of extract DNA per hour. The lower limit of quantitation (LLOQ) was defined as 2.3 Fmoles ethenoadenine removed per *μ*g DNA per hour.

### *N*7-methylguanine in DNA

The amounts of 7-meG in peripheral blood DNA were quantified in pre- and post-DTIC treatment (day 1 at 5 h and day 8) samples according to the immunoblot method previously described (Menear *et al*, 2008). The chemiluminescence signal was generated by incubation with ECL-Advance (Amersham, Chalfont St Giles, UK), detected with the Chemi Genius Bio Imaging System (Syngene, Cambridge, UK) and quantified using Genetools software (Syngene). The LLOQ was defined as 0.3 Fmoles 7-meG per *μ*g DNA.

### Pharmacokinetics

Venous blood was drawn for determination of pharmacokinetic profiles for olaparib and dacarbazine when dosed alone and in combination. Blood samples were collected before the patients started taking olaparib and 0.25, 0.5, 1, 1.5, 2, 3, 4, 6, 8 and 9–10 h after taking the drug on day 1 of cycle 1. Blood samples were also collected before the start of the dacarbazine infusion and 0.5, 1, 2 and 5–6 h after the end of infusion to quantify plasma levels of dacarbazine on day 1 of cycles 1 and 2. Plasma dacarbazine and olaparib concentrations were determined by high-performance liquid chromatography with tandem mass spectrometric detection.

## Results

A total of 40 patients were enroled in the study at three centres, and their characteristics are summarised in Tables 1 and 2. All 40 patients were evaluable for toxicity and for tumour response.

### Dose escalation and extent of exposure

A total of 153 cycles (median 2.5 cycles per patient) of olaparib and dacarbazine were administered in the study. There were no dose reductions of olaparib, although six patients were non-compliant and missed one dose of their olaparib treatment each. Four patients required dacarbazine dose reductions of 200 mg m^−2^ and in one patient, a second dose reduction was needed because of haematological toxicity.

During the dose-escalation phase of the trial, three cohorts of patients were successfully treated without major potentiation of toxicity or serious side effects. When patients were treated with 40: 800 in part II, a higher incidence of bone marrow toxicity, particularly neutropaenia, was noted compared with rates reported for single-agent DTIC. Although the toxicities were not dose limiting per protocol, it was apparent that the dose combination of 40: 800 could not be sustained over several cycles. Therefore, the protocol was amended to allow a dose combination of 600 mg m^−2^ DTIC in combination with increasing olaparib doses from 40 to 200 mg bd to be explored.

Three patients in the dose-escalation phase experienced a DLT during the first treatment cycle. One occurred in cohort 4 (40: 800) and two in cohort 6 (200: 600). The highest combination doses that were deliverable were 100: 600 and 20: 800. Maximal PARP inhibition was observed at doses above 60 mg bd in single-agent trials so the 100: 600 dose combination was chosen for the dose-expansion component of the study (Fong *et al*, 2009).

### Safety

Adverse events were as anticipated for dacarbazine (Table 3). Most frequent toxicities were neutropaenia and anaemia, although nausea, fatigue, anorexia, diarrhoea and thrombocytopaenia were common. As anticipated, the incidence of neutropaenia was higher than that observed with single-agent dacarbazine, affecting 10 patients and in 9 of these, the severity was ⩾grade 3. Two patients died within 30 days of receiving study drug. Neither death was considered to be related to the combination treatment: one because of myocardial ischaemia and the other to pneumonia in the setting of progressive disease.

### Dose-limiting toxicities

One patient treated with 40: 800 experienced grade 3 hypophosphataemia and grade 3 leucopoenia. Cycle 2 was delayed because of thrombocytopaenia, which peaked at grade 3 and did not recover until day 49. The patient was taken off study because of these toxicities. Two patients treated with 200: 600 experienced grade 4 neutropaenia on day 15, recovering by day 22. This was considered to be dose limiting, although the duration of neutropaenia could not be confirmed. These two dose levels were not chosen for further evaluation because of the toxicities observed in these cohorts, in particular the occurrence of neutropaenia in one-third of patients in each of the cohorts with insufficient recovery before the date of the next planned dacarbazine infusion.

### Efficacy

All patients were evaluable for efficacy. Two partial responses were documented, and eight patients had stable disease where follow-up measurements must have met the stable disease criteria at least once after trial entry at a minimum interval of 12 weeks. The other 30 patients progressed on treatment (Table 4). Both partial responses were seen in patients with previously treated melanoma: one where the patient had received five previous lines of therapy and the other where they had received one previous regimen.

The median time to disease progression was 82 days (95% confidence interval (CI): 38–108 days) in the dose-escalation phase in refractory solid tumour patients and 42 days (95% CI: 36–84 days) for chemotherapy-naive melanoma patients. The median time to disease progression overall was 43 days (95% CI: 36–108 days).

### Pharmacokinetics

A total of 29 patients provided evaluable pharmacokinetic data for olaparib with or without dacarbazine. The co-administration of dacarbazine appeared to have had little or no effect on exposure to olaparib, with respect to peak concentration (*C*_max_) or area under the concentration–time curve. Similarly, in 17 evaluable patients, there was no discernable impact of olaparib on dacarbazine pharmacokinetics.

### Pharmacodynamics

**Figure 2:**
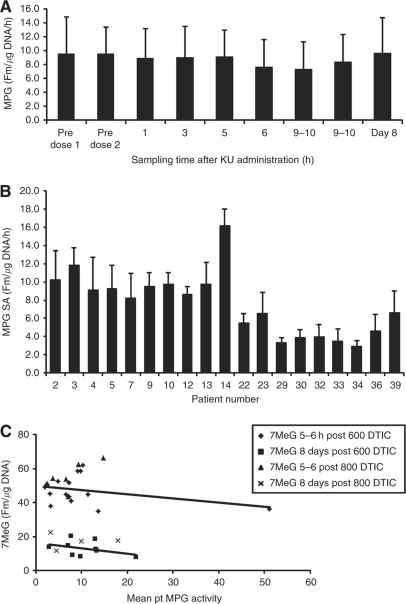
Pharmacokinetic data: (**A**) Activity of *N-*methylpurine-DNA glycosylase (MPG) during course of sampling after olaparib administration. (**B**) Mean MPG activity for 21 available patients. (**C**) Effect of MPG activity on 7-methylguanine (7-meG) levels.

Blood samples collected from 24 patients were available for analysis. For individual patients, MPG activities did not vary significantly or consistently over the course of the sampling (Figure 2A), indicating that MPG activity was not substantially affected by the doses and schedules of olaparib and dacarbazine used in this study, or sampling times. Mean MPG activity, based on all times points measured, showed marked interpatient variation, with values ranging from 3.3 to 16.2 Fmol per *μ*g DNA per hour (mean±s.d. 8.7±6.0; Figure 2B).

Levels of 7-meG before treatment were less than the LLOQ for 14 out of the 20 patients analysed, with very low levels in the other 6 individuals (range 0.3–1.6 Fmol per *μ*g DNA). Mean 7-meG levels rose significantly (*P*<0.01) on day 1, 5 h after dacarbazine administration (50.5±8.2 Fmol per *μ*g DNA, *n*=24), and remained above baseline at day 8 (14.6±5.4 Fmol per *μ*g DNA, *n*=5). This is consistent with previous observations made following temozolomide treatment (Watson *et al*, 2009) that levels reduce over time, most likely because of cell turnover and/or removal of 7-meG by MPG. Mean 7-meG levels after treatment were higher in patients treated with 800 mg m^−2^ dacarbazine (55.3±8.1 Fmol per *μ*g DNA) than those treated with 600 mg m^−2^ (47.4±8.3 Fmol per *μ*g DNA) (*P*<0.05). There was no correlation between MPG activity and 7-meG levels after dacarbazine administration in individual patients (Figure 2C). Unfortunately, no data were available from the two patients who had partial responses on treatment.

## Discussion

Treatment options for patients with metastatic melanoma are limited, with existing therapies only producing low rates of objective radiological responses and short periods of clinical benefit. Dacarbazine remains the standard of care and is the benchmark against which other therapies are compared.

This is the first study evaluating the optimally tolerated dose of olaparib, a potent PARP inhibitor, in combination with dacarbazine in patients with either advanced solid tumours or chemotherapy-naive melanomas. Adverse event rates were similar to those previously reported for dacarbazine alone at doses from 800 to 1000 mg m^−2^, with the exception of neutropaenia. Myelosuppression occurs in about 25% of patients treated with dacarbazine, but grade 3 or 4 events are seen in only 1–2% (Bajetta *et al*, 1994; Middleton *et al*, 2000). As expected from other studies with PARP inhibitors in combination with temozolomide and pre-clinical studies with olaparib significant myelotoxicity, especially neutropaenia was more frequent and defined the dose of the two drugs for combined use. Grade 3 or 4 neutropaenia affected nine patients (22.5%) in this study, and appeared more frequent with higher doses of dacarbazine or olaparib. The increased neutropaenia, although readily managed, led to an increase in delays in the administration of the second cycle of therapy and reductions in the dose of dacarbazine in the highest dose cohorts.

The 100 mg bd olaparib and 600 mg m^−2^ dacarbazine dose combination was chosen for the dose confirmation component of the study. Monotherapy olaparib studies had indicated that doses of 100 mg bd or above were clinically efficacious and inhibited PARP-1 (Fong *et al*, 2009), so this combination was preferred over one with a higher dacarbazine dose and 20 mg bd olaparib. Although dose modifications were required for some patients, this dose was generally well tolerated in chemonaive melanoma patients, confirming its suitability for use outside of a clinical trial setting. Experience of myelosupression with PARP inhibitors and methylating agents to date has been variable. Use of AG-14699 required a reduction in the dose of temozolomide given concurrently, as was the case in the current study. However, full-dose temozolomide can be administered with a PARP-inhibitory dose of veliparib (ABT-888). The basis for this difference in the enhancement of myelotoxicity is not clear, as all the drugs are potent PARP-1 inhibitors.

The dose confirmation cohort was also designed to give a preliminary assessment of the efficacy of this dose combination, before potentially expanding recruitment to compare combination therapy with dacarbazine alone. The minimum efficacy requirement to move to the randomised phase of the trial was an objective response rate of 20% or greater after four cycles of treatment. As there were no further responses in the 10 melanoma patients treated first line with the combination, this requirement was not met and the expansion phase of the study was not opened. Progression-free survival was similar to that obtained with dacarbazine and temozolomide in previous clinical trials (Eigentler *et al*, 2003; Quirt *et al*, 2007).Given the limited numbers of chemotherapy-naive melanoma patients in this study, the possibility of a small benefit for the addition of the PARP inhibitor in this group of patients cannot be excluded.

The MPG activity was detectable in all samples analysed, showed considerable interpatient variation (Figure 2A) but was not significantly affected by administration of either olaparib or dacarbazine or sampling time (Figure 2B). It was anticipated that MPG activity might influence 7-meG levels, but this proved not to be the case (Figure 2C). As dacarbazine requires metabolic activation for conversion to a methylating species, interpatient differences in metabolism will influence initial methylation levels, potentially confounding any correlation between MPG activity and 7-meG. However, mean 7-meG levels measured 5 h after dacarbazine administration were remarkably consistent, so this is unlikely to explain the lack of correlation. Thus, in peripheral blood mononuclear cells, the availability of functional MPG is not the principal determining factor in the loss of 7-meG. Although there are no reports of other repair processes acting on this lesion, it is possible that factors such as MPG recruitment to damage site, cofactors and coupling to transcription-coupled or global genome repair processes, may modulate 7-meG removal.

A phase I study of AG014699 with temozolomide in patients with metastatic melanoma was the first clinical trial of a PARP inhibitor in combination with chemotherapy (Plummer *et al*, 2008). The combination was well tolerated, with some evidence of activity. However, in a phase II study of the combination, the haematological toxicity of temozolomide was exacerbated with one toxic death, three neutropaenic hospitalisations and dose reductions of temozolomide in a significant proportion of patients, highlighting the differences that can occur, as regimens identified in select populations in phase I trials are applied more widely. The level of PARP inhibitor activity has not been reported (Plummer *et al*, 2006). Other PARP inhibitors have been tested in melanoma in combination with a variety of cytotoxic or targeted agents. By far the largest study of this approach is the recently completed randomised phase 2 trial of ABT-888 and temozolomide. This trial of over 300 patients will provide the clearest insight into the potential for PARP inhibition in melanoma when its results become known late in 2010.

In conclusion, this phase I study identified clinically tolerable doses of olaparib and dacarbazine for use in combination therapy in patients with metastatic melanoma. However, no responses were seen in 10 chemotherapy-naive melanoma patients treated at the optimal combination doses identified. Despite the small size of this group, it is unlikely that this combination, at these doses, will provide a clinically important advantage over single-agent dacarbazine as first-line treatment in patients with advanced melanoma.

## Figures and Tables

**Table 1:** Patient demographics

Number of patients	40
Mean age, years (range)	53.3 (19–75)
Gender (male/female)	28/12
Performance status (0/1/2)	23/15/2
	
*Tumour type (*n*)*	
Melanoma	33
Colorectal	2
Transitional cell carcinoma bladder	1
Squamous cell carcinoma vulva	1
Haemangioblastoma	1
Merkel cell carcinoma	1
Adenocarcinoma lung	1
	
*Previous chemotherapy regimens (*n*)*	
0	21
1	7
2	7
⩾3	5

**Table 2:** Disposition of patients across different cohorts

**Baseline characteristics**								
	**10/600 (*n*=3)**	**20/600 (*n*=4)**	**20/800 (*n*=4)**	**40/600 (*n*=4)**	**40/800 (*n*=6)**	**100/600 (*n*=13)**	**200/600 (*n*=6)**	**Total (*n*=40)**
Refractory solid tumour, *n* (%)	3 (100)	4 (100)	0	4 (100)	0	3 (23.1)	6 (100)	20 (50)
First-line melanoma, *n* (%)	0	0	4 (100)	0	6 (100)	10 (76.9)	0	20 (50)
Malignant melanoma, NOS, *n* (%)	3 (100)	1 (25)	4 (100)	1 (25)	6 (100)	12 (92.3)	3 (50)	30 (75)
Other, *n* (%)	0	3 (75)	0	3 (75)	0	1 (7.7)	3 (50)	10 (25)

Abbreviation: NOS=not otherwise specified.

**Table 3:** Summary of number (%) of patients with adverse events of grade 3 or higher, occurring in at least 5% of patients

	**Initial dose level (olaparib mg bd per DTIC mg m^−2^)**							
**Number of patients with AE** **(%)**	**10/600 (*n*=3)**	**20/600 (*n*=4)**	**20/800 (*n*=4)**	**40/600 (*n*=4)**	**40/800 (*n*=6)**	**100/600 (*n*=13)**	**200/600 (*n*=6)**	**Total overall (*n*=40)**
Patients with any grade 3 or higher adverse events	2 (66.7)	4 (100)	1 (25.0)	2 (50.0)	5 (83.3)	9 (69.2)	6 (100)	29 (72.5)
								
*Blood and lymphatic system*								
Anaemia	0	1 (25.0)	0	0	0	0	1 (16.7)	2 (5.0)
Leucopoenia	0	1 (25.0)	0	0	1 (16.7)	1 (7.7)	2 (33.3)	5 (12.5)
Lymphopaenia	0	1 (25.0)	0	0	1 (16.7)	2 (15.4)	2 (33.3)	6 (15.0)
Neutropaenia	0	1 (25.0)	0	0	3 (50.0)	3 (23.1)	2 (33.3)	9 (22.5)
Thrombocytopaenia	0	0	0	0	1 (16.7)	0	2 (33.3)	3 (7.5)
								
*Gastrointestinal*								
Abdominal pain upper	0	0	0	0	1 (16.7)	1 (7.7)	0	2 (5.0)
								
*Metabolism and nutrition*								
Hyperglycaemia	0	0	0	1 (25.0)	0	1 (7.7)	0	2 (5.0)
Hyponatraemia	1 (33.3)	1 (25.0)	0	0	0	0	0	2 (5.0)
Hypophosphataemia	1 (33.3)	1 (25.0)	0	0	1 (16.7)	1 (7.7)	0	4 (10.0)
								
*Musculoskeletal and connective tissue*								
Arthralgia	0	0	0	1 (25.0)	1 (16.7)	0	0	2 (5.0)
Back pain	0	0	0	0	1 (16.7)	1 (7.7)	0	2 (5.0)
								
*Nervous system*								
Lethargy	0	0	1 (25.0)	0	0	1 (7.7)	0	2 (5.0)

Abbreviations: AE=adverse events; CTCAE=Comman Terminology Criteria for Adverse Events, CTCAE grade 3=severe; 4=life threatening or disabling; DTIC=dimethyltriazenoimidazole carboxamide.

**Table 4:** Overall best response summary (RECIST): intent-to-treat population

	**Initial dose level (olaparib mg bd per DTIC mg m^−2^)**							
**Overall best response, *n* (%)**	**10/600 (*n*=3)**	**20/600 (*n*=4)**	**20/800 (*n*=4)**	**40/600 (*n*=4)**	**40/800 (*n*=6)**	**100/600 (*n*=13)**	**200/600 (*n*=6)**	**Total (*n*=40)**
Partial response	0	0	0	1 (25.0)	0	0	1 (16.7)	2 (5.0)
Progressive disease	3 (100)	3 (75.0)	4 (100)	3 (75.0)	5 (80.0)	9 (69.2)	3 (50.0)	30 (75.0)
Stable disease	0	1 (25)	0	0	1 (20.0)	4 (30.8)	2 (33.3)	8 (20.0)

Abbreviation: RECIST=response evaluation criteria in solid tumors.
